# Enhancing precision flood mapping: Pahang’s vulnerability unveiled

**DOI:** 10.1371/journal.pone.0310435

**Published:** 2024-11-07

**Authors:** Tahmina Afrose Keya, Siventhiran S. Balakrishnan, Maheswaran Solayappan, Saravana Selvan Dheena Dhayalan, Sreeramanan Subramaniam, Low Jun An, Anthony Leela, Kevin Fernandez, Prahan Kumar, A. Lokeshmaran, Abhijit Vinodrao Boratne, Mohd Tajuddin Abdullah

**Affiliations:** 1 Department of Community Medicine, AIMST University, Bedong, Kedah, Malaysia; 2 Department of Community Medicine, MGMCRI, Sri Balaji Vidyapeeth (Deemed–to be-University), Pondicherry, India; 3 Department of Research Management Centre, AIMST University, Bedong, Kedah, Malaysia; 4 Department of Applied Sciences, AIMST University, Bedong, Kedah, Malaysia; 5 Department of Engineering & Computer Technology, AIMST University, Bedong, Kedah, Malaysia; 6 Centre for Chemical Biology, Universiti Sains Malaysia (USM), Bayan Lepas, Penang, Malaysia; 7 School of Biological Sciences, Universiti Sains Malaysia (USM), Georgetown, Penang, Malaysia; 8 Department of Medical Microbiology, AIMST University, Bedong, Kedah, Malaysia; 9 Department of Community & Family Medicine, AIIMS Deoghar, Jharkhand, India; 10 Academy of Science Malaysia, Jalan Sultan Haji Ahmad Shah, Kuala Lumpur, Malaysia; Apeejay Stya University, INDIA

## Abstract

Malaysia, particularly Pahang, experiences devastating floods annually, causing significant damage. The objective of the research was to create a flood susceptibility map for the designated area by employing an Ensemble Machine Learning (EML) algorithm based on geographic information system (GIS). By analyzing nine key factors from a geospatial database, flood susceptibility map was created with the ArcGIS software (ESRI ArcGIS Pro v3.0.1 x64). The Random Forest (RF) model was employed in this study to categorize the study area into distinct flood susceptibility classes. The Feature selection (FS) method was used to ranking the flood influencing factors. To validate the flood susceptibility models, standard statistical measures and the Area Under the Curve (AUC) were employed. The FS ranking demonstrated that the primary attributes to flooding in the study region are rainfall and elevation, with slope, geology, curvature, flow accumulation, flow direction, distance from the river, and land use/land cover (LULC) patterns ranking subsequently. The categories of ’very high’ and ’high’ class collectively made up 37.1% and 26.3% of the total area, respectively. The flood vulnerability assessment of Pahang found that the Eastern, Southern, and central regions were at high risk of flooding due to intense precipitation, low-lying topography with steep inclines, proximity to the shoreline and rivers, and abundant flooded vegetation, crops, urban areas, bare ground, and rangeland. Conversely, areas with dense tree canopies or forests were less susceptible to flooding in this research area. The ROC analysis demonstrated strong performance on the validation datasets, with an AUC value of >0.73 and accuracy scores exceeding 0.71. Research on flood susceptibility mapping can enhance risk reduction strategies and improve flood management in vulnerable areas. Technological advancements and expertise provide opportunities for more sophisticated methods, leading to better prepared and resilient communities.

## Introduction

A disaster is an event of considerable magnitude that surpasses the capabilities of local resources, thereby requiring external aid [1]. Floods are a frequently occurring natural disaster that can lead to widespread illness and loss of life on a global scale. The extent of damage to communities is influenced by factors such as geography, population density, and infrastructure [2]. The occurrence of hydrometeorological disasters such as floods, heavy rainfall, and tropical storms has been linked to climate change [3]. In Malaysia, particularly in its eastern region, the annual monsoon floods pose significant dangers to individuals residing near riverbanks [4,5].

Malaysia is characterized by a tropical climate, featuring high temperatures, humidity, and abundant rainfall. The country experiences distinct monsoon seasons that impact both the peninsular and insular regions. The Northeast Monsoon, occurring from November to March, brings heavy rainfall and rough seas, often leading to flooding in the eastern part of the peninsula. On the other hand, the Southwest Monsoon dominates from May to September, primarily affecting the southwestern coastal areas of Sabah. The First and Second Inter-monsoonal Periods fall in between these two main monsoon seasons. Malaysia receives significant annual rainfall, with Maxwell’s Hill recording the highest amount at 5,000 mm [6–9]. Pahang, the third largest state in Malaysia, is located in the basin of the Pahang River, extending from the east coast to Endau. Positioned in the East Coast region of Malaysia, it spans an area of 35,965 km^2^. Pahang showcases a tropical landscape and encounters an equatorial climate marked by elevated humidity all year round. The coastal area of Pahang in Peninsular Malaysia is significantly affected by strong northerly winds and intense rainfall during the early and mid-monsoon period, resulting in severe floods from November to January. In contrast to other regions in Peninsular Malaysia, the east-coast section of Pahang receives higher annual precipitation levels with more pronounced variations. Pahang has witnessed significant flood occurrences that inflicted substantial damage on the state, commencing with the catastrophic flood event in 1926, which was documented as the most severe flood incident in history. The year 2014 witnessed one of the most devastating flood events in Pahang and the entire East Coast of Peninsular Malaysia. The major flood event in Pahang in 2014 caused extensive damage, particularly to the local economy and alterations in the hydrological system. Notably, Pahang encountered major floods in 1971 and 2021, resulting in widespread destruction and influencing both the local economy and hydrological system [8,10].

Ensemble learning in machine learning is a powerful technique that improves forecasting accuracy by aggregating predictions from multiple models to minimize errors and biases. Various advanced Ensemble Machine Learning (EML) methods exist, such as Stacking, Blending, Bagging, Boosting, and Random Forest. The utilization of GIS-based machine learning algorithms to construct models for forecasting natural calamities in particular areas has witnessed a surge. Ensemble Machine Learning (EML) amalgamates numerous classifiers to enhance accuracy, resulting in more precise predictions compared to the utilization of a single classifier. The incorporation of ensemble models strives to diminish prediction errors and enhance overall accuracy [11–15]. Hence, the overarching goal of this research is to develop a comprehensive flood susceptibility mapping framework for the Pahang State, Malaysia using a Geographic Information System (GIS) and EML approach. This framework aims to enhance disaster preparedness, inform land use planning, and mitigate the impacts of recurrent floods on communities and infrastructure in the region.

The research aimed to develop a robust GIS-based framework for flood susceptibility mapping in Pahang state, utilize ensemble machine learning algorithms to build predictive flood susceptibility models, and provide actionable insights for decision-making and disaster management. This includes identifying at-risk areas, analyzing factors affecting flood risk, and recommending specific interventions and mitigation strategies to reduce flood impacts. The Pahang region in Malaysia has experienced frequent floods, causing significant challenges to the community, infrastructure, and economy. Despite efforts to mitigate flood risks, there is a need for accurate and comprehensive flood vulnerability mapping. Conventional techniques often lack precision and fail to consider environmental factors and the dynamic nature of climate change and land use patterns. This research aimed to address these limitations and inform decision-making and disaster management strategies. The research is expected to forecast that the combination of these flood influencing factors will collectively serve as the main determinants of flood susceptibility in Pahang. It is anticipated that the EML method, particularly the Random Forest-embedding model, will effectively capture the intricate relationships among these factors to generate precise flood susceptibility forecasts. Moreover, the study will posit that the identified susceptibility categories, spanning from very low to very high, will offer a dependable depiction of the diverse levels of flood vulnerability in different areas of Pahang. The validation procedure, which involves Area Under the Curve (AUC) analysis, is also provided a robust evaluation of the model’s predictive accuracy, thereby bolstering confidence in the resultant flood susceptibility map for Pahang.

## Methods

The laboratory protocol of this study was deposited and published to the protocols.io (Protocol ID #101308, DOI: dx.doi.org/10.17504/protocols.io.kxygxyy6zl8j/v1) [16].

### Study area

Pahang, located in Peninsular Malaysia, was selected as the study area because of its yearly monsoon floods, which have a detrimental impact on the local community.

### Study design and data collection tool

#### Flood influencing factors

According to the data available for Pahang and a comprehensive literature search, a total of nine factors have been identified as potential indicators of heightened flood susceptibility in the context of modelling studies. These factors encompass elevation, slope, curvature, flow direction, flow accumulation, distance from river, rainfall, land-use, and geology. Together, these parameters effectively capture the topographical and hydrometeorological conditions that contribute to the overall vulnerability of the region to flooding events [17,18].

**Fig 1:**
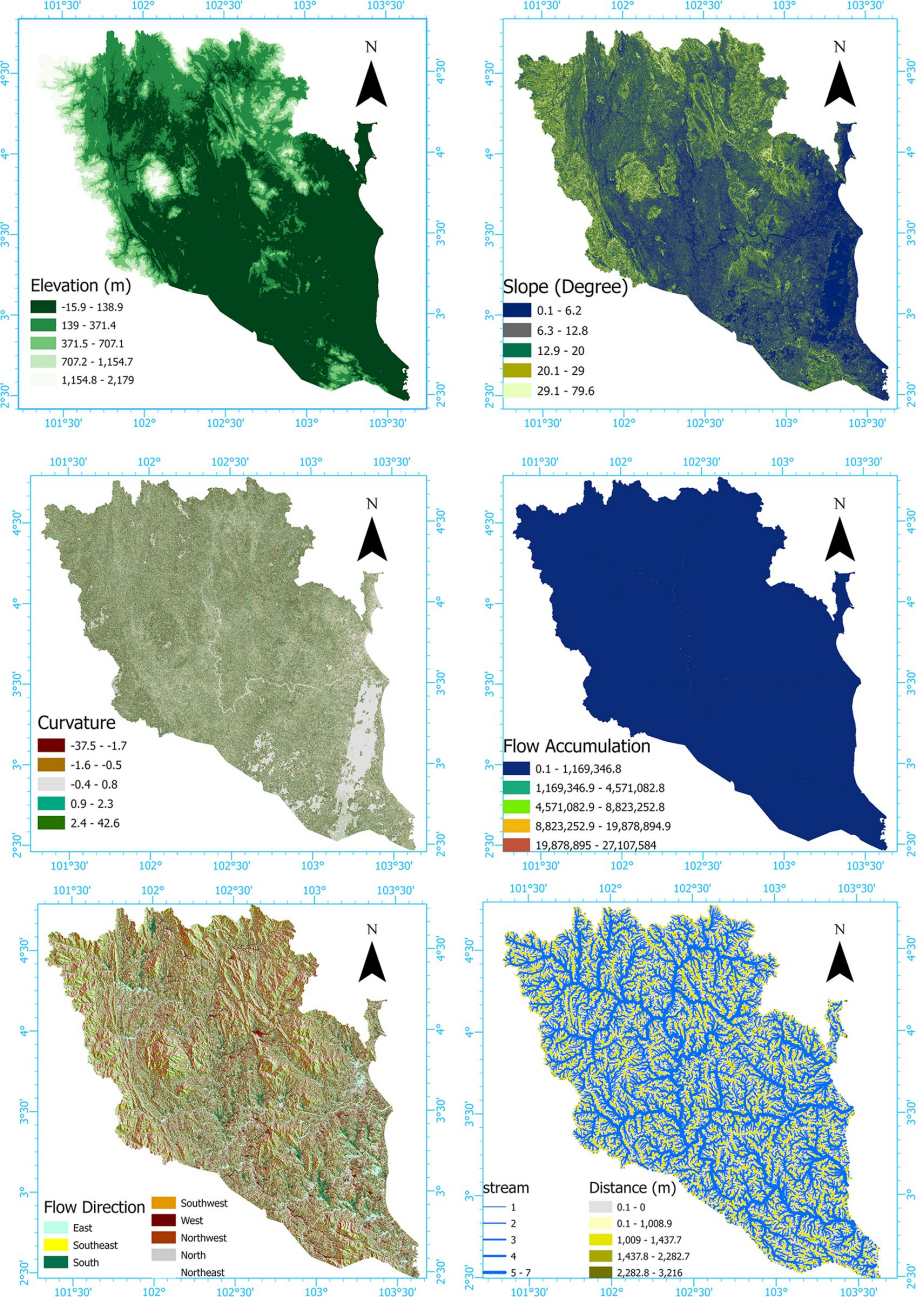
Flood conditioning factor maps: Elevation, slope, curvature, flow-direction, flow-direction. Distance. (This map has been created by the author using ArcGIS Software: ESRI ArcGIS Pro v3.0.1 x64).

Digital Elevation Models (DEMs) have demonstrated their indispensable role in ensuring the precision of hydrodynamic models [19]. The Earth data platform provided access to the 30 m resolution Shuttle Radar Topography Mission (SRTM) DEM Version 3, from which the digital elevation data was obtained [20]. The DEM of the study area was utilized in ArcGIS Software (ESRI ArcGIS Pro v3.0.1 x64) to generate the elevation map. To classify the elevation map, the natural break classification method was employed, resulting in five distinct class intervals for the study. The presence of flooding is largely impacted by the slope of the land, as steeper slopes can accelerate the flow of water over the surface, hindering its ability to seep into the ground [21]. The DEM map was used to generate the slope angle map in ArcGIS Software (ESRI ArcGIS Pro v3.0.1 x64), which was then categorized into five classes using the natural break classification method. The slope angle map of the study area exhibits a range of 0.5° to 79.6°. The shape of a surface, as determined by its curvature, indicates whether it is convex, concave, or flat, indicating changes in slope inclination. Concave surfaces tend to collect flood water, increasing the likelihood of flooding [22]. The direction of flow plays a crucial role in determining the path that surface water will take and the potential for flooding [23]. The digital elevation model (DEM) was utilized for producing the curvature map within the ArcGIS Software (ESRI ArcGIS Pro v3.0.1 x64). Subsequently, the curvature map was divided into five distinct classes through the application of the natural break classification technique. The curvature map of the specific research site displays a variation from -37.5 to 42.6 degrees. Flow direction is a hydrological parameter indicating the path in which water flows [23]. A flow direction map was generated utilizing the Arc Hydro Tool (ESRI ArcGIS Pro v3.0.1 x64). This map was then categorized into eight distinct classes, representing the various directions in which water can flow. These classes include east, southeast, south, southwest, west, northwest, north, and northeast direction. An increase in flow accumulation coincides with an increase in vulnerability to flooding [21]. The flow accumulation map was generated by the utilization of the flow accumulation tool within the ArcGIS software (ESRI ArcGIS Pro v3.0.1 x64), which processed the flow direction map. This particular tool computed the cumulative flow within each upstream cell, directing it towards the downslope cells in the resulting raster image. The estimation of the distance from rivers was conducted in this study by employing the Euclidean distance tool within the ArcGIS software (ESRI ArcGIS Pro v3.0.1 x64). This tool made use of a raster layer that represented the river network. Subsequently, the distance from the river was categorized into five classes using the geometric interval classification method. These classes spanned from 0.1 meter to 3,216 meters (Fig 1) and S1 File.

**Fig 2:**
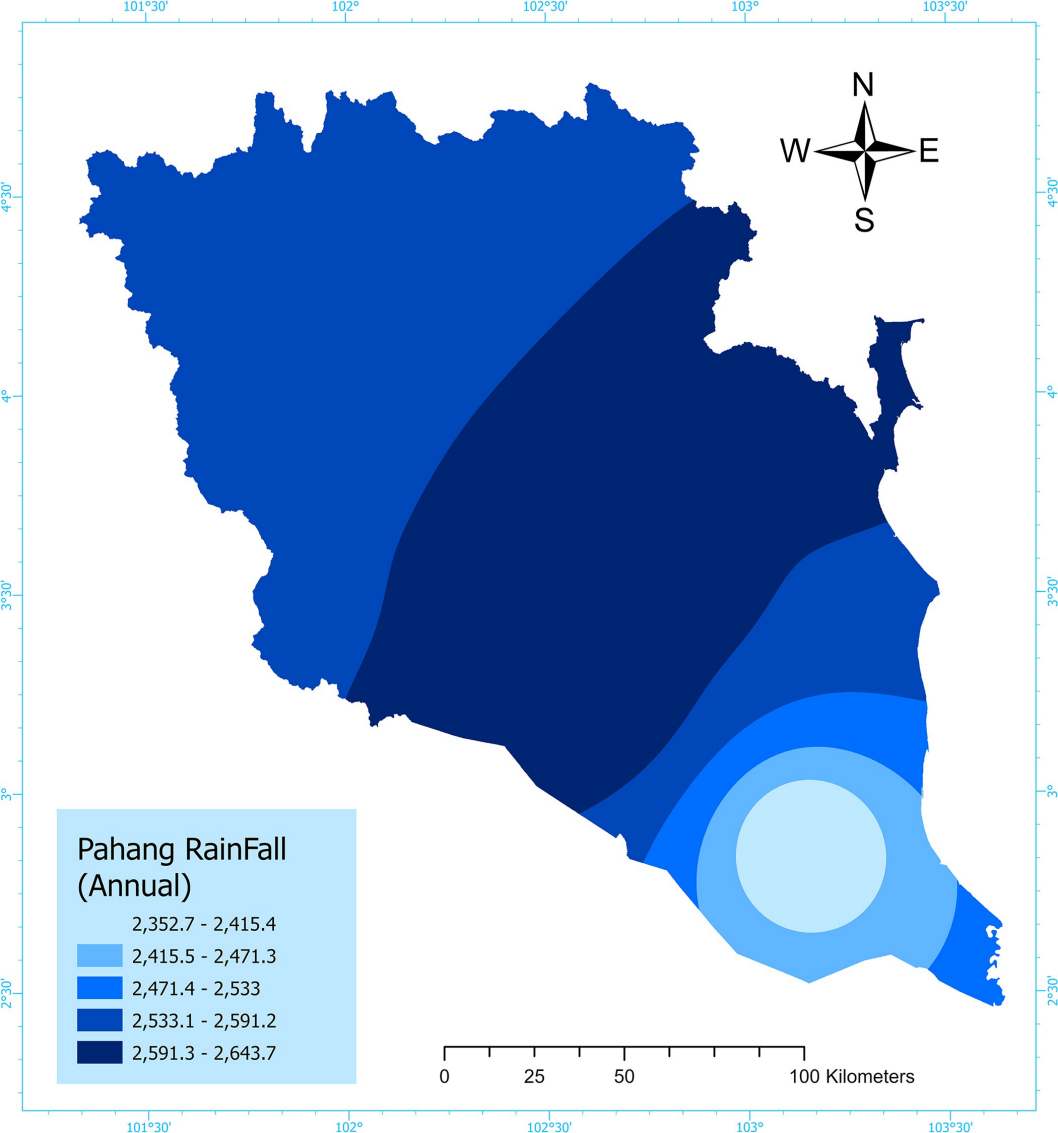
Flood conditioning factor map: Rainfall. (This map has been created by the author using ArcGIS Software: ESRI ArcGIS Pro v3.0.1 x64).

Flooding occurs when there is a sudden increase in water levels in rivers, lakes, and reservoirs due to intense rainfall, often resulting in inadequate drainage [24]. A rainfall distribution map for the research area was constructed using ArcGIS Software (ESRI ArcGIS Pro v3.0.1 x64). Data from 10 precipitation stations in Pahang, namely Cameron Highlands, Bentong, Bera, Kuantan, Lipis, Maran, Pekan, Raub, Rompin, and Temerloh, were utilized. The Inverse Distance Weighted (IDW) approach was employed, incorporating a 10-year dataset from 2012 to 2021 [25]. The IDW technique guaranteed the precise representation of rainfall distribution in the specific research area. The IDW approach accurately illustrated the rainfall patterns within the study area [26] (Fig 2) and S1 Dataset.

**Fig 3:**
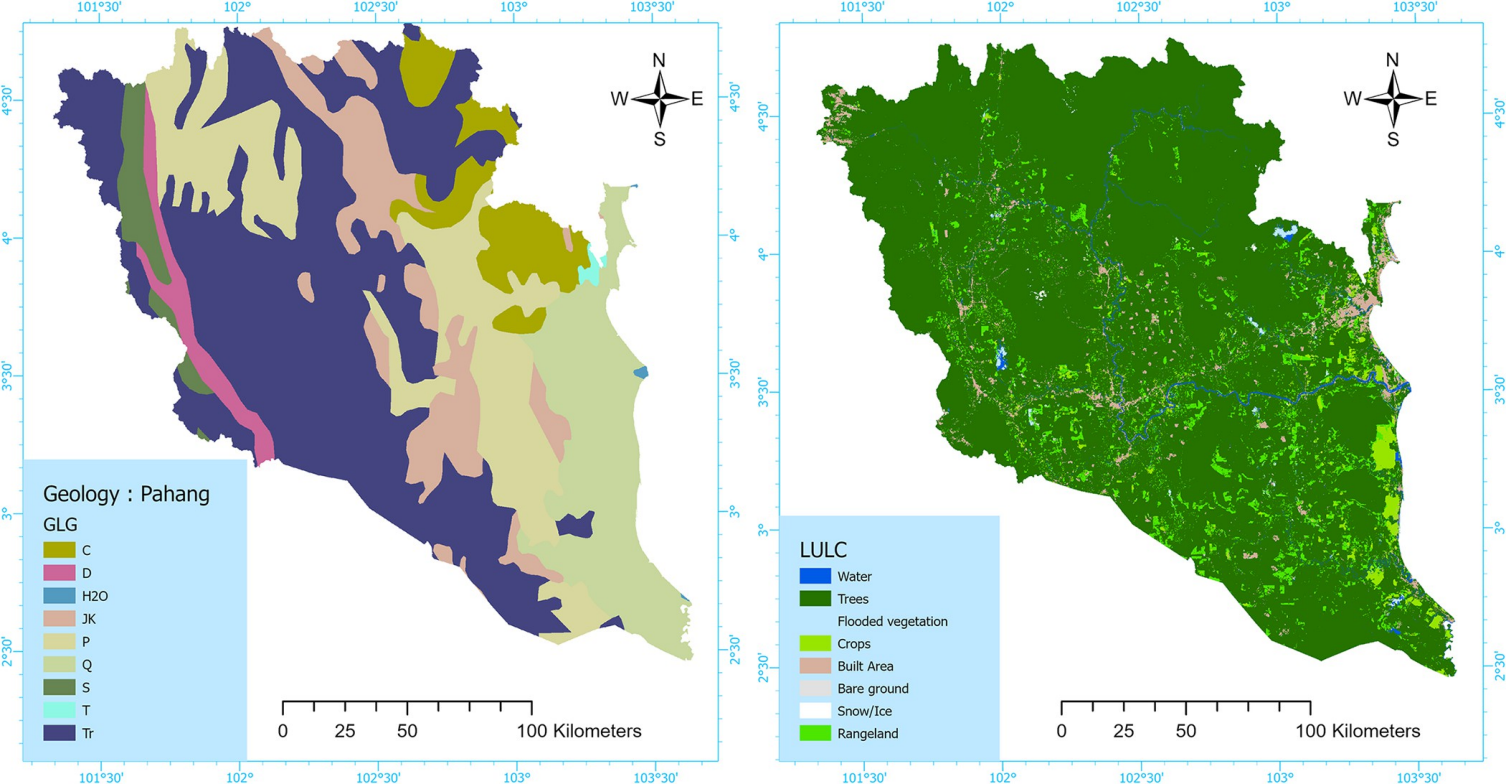
Flood conditioning factor maps: LULC and geology. (This map has been created by the author using ArcGIS Software: ESRI ArcGIS Pro v3.0.1 x64).

The properties of drainage systems are significantly affected by changes in land use and land cover (LULC) in the upstream watersheds. These modifications directly impact the occurrence of surface overflow and the land surface’s capacity to absorb water, ultimately playing a role in the frequency and intensity of flooding events [27]. The global geological and LULC data were obtained from the worldwide geological maps database provided by the USGS and the Global Data [28]. The LULC map was created using the ArcGIS Software (ESRI ArcGIS Pro v3.0.1 x64), delineating seven distinct categories: water bodies, trees, flooded vegetation, crops, built area, bare terrain, and rangeland. The geologic map of Pahang was generated utilizing the ArcGIS Software (ESRI ArcGIS Pro v3.0.1 x64) and is divided into nine primary soil features, which include Carboniferous (C), Devonian (undivided) (D), Water bodies (H2O), Jurassic/Cretaceous (JK), Permian (P), Quaternary (Q), Silurian (S), Tertiary (T) (undivided), and Triassic (Tr) based on the USGS-USA soil taxonomy [29]—(Fig 3) and S1 File.

### Ensemble machine learning methods (EMLs)

Ensemble methods are designed to enhance the performance and reliability of a model by creating multiple models and integrating their predictions. By combining the outputs of these models, ensemble methods aim to improve the robustness and generalizability of the overall model. Various advanced Ensemble Machine Learning (EML) methods exist, such as Bagging, Boosting, and Random Forest [13,14].

**Bagging:** Bootstrapping balances representation in base models by running them on subsets of the dataset called bags, which are subsets with replacement instances to match the original dataset size. The result is obtained by merging the outputs of all base models [30].

**Boosting:** Boosting is a method that enhances a weak base model by creating a new model that corrects errors made by previous models, resulting in a strong learner through a weighted average of all models. AdaBoost trains weak learners on iteratively adjusted versions of the dataset. Gradient Tree Boosting, or GBRT, is a versatile boosting technique that can be used with differentiable loss functions for regression and classification tasks. It is reliable and efficient [14].

**Random forest (RF)/Random Trees Embedding classifier:** Within random forests, specifically in the Random Forest Classifier and Random Forest Regressor classes, every tree within the collection is constructed using a sample that is selected with replacement, known as a bootstrap sample, from the training dataset. Random Trees Embedding utilizes a collection of arbitrarily selected trees to represent the data through the leaf indices where a data point is located. These indices are transformed into a one-of-K encoding, resulting in a sparse binary coding with high dimensionality. The computation of this coding is highly efficient and serves as a foundation for various learning activities. The dimensions and sparsity of the code can be adjusted by specifying the quantity of trees and the maximum depth for each tree. Random Forest employs bagging and extends the bagging estimator algorithm. In Random Forest, decision trees serve as the base estimators, and a random selection of features is used to determine the best split at each node of the decision tree. The random forest technique demonstrates strong predictive accuracy and is adept at managing large datasets for regression and classification purposes. By training numerous decision trees concurrently through bootstrapping, aggregation, and bagging methods, the RF method consistently outperforms alternative techniques in accuracy and prevents overfitting. Moreover, the training process for the RF-embedding model is quicker, leading to superior classification accuracy [31,32]. The RF model demonstrated superior accuracy (0.89) in our study when compared to both bagging and Boosting Classifiers, highlighting the robust predictive capabilities of the RF model (Fig 8).

ArcGIS Software (ESRI ArcGIS Pro v3.0.1 x64) for creating and analyzing the maps, anaconda navigator (anaconda3), Colab for EML algorithms, and R software (R v4.3.2) were employed as tools for data analysis. The relationship between flood influencing factors and flood susceptibility was investigated using logistic regression analysis. A p-value of less than 0.001 was deemed statistically significant.

### Ethical clearance

Our research study has been granted ethical approval by both the Research Management Centre (RMC) Committee [Application Ref No: AIMST/RMC/AUHAEC/FRGS/25022022/01, Date: 25/02/2022] and the AIMST University Human Ethics Committee (AUHEC) [Application Ref No: AUHEC/FOM/22/09/2023/, Date: 22/09/2023].

## Results

### The flood influencing factors

In this study, nine factors have been identified as potential indicators of increased vulnerability to flooding in modelling studies. These factors include elevation, slope, curvature, flow direction, flow accumulation, distance from the river, rainfall, land-use, and geology. Table 1 illustrates the utilization of logistic regression analysis to examine the relation between the factors influencing floods and the susceptibility to flooding. A p-value of less than 0.001 was deemed statistically significant “Table 1”.

**Table 1:** Logistic regression model (coefficients)–flood susceptibility.

95% Confidence Interval						
Flood Predictors	Estimate	SE	p	Odds ratio	Lower	Upper
**Intercept**	-1.82	0.29	< .001	0.2	0.1	0.3
**Rainfall (mm/year)**						
**2352.7–2415.4(Ref.)**						
**2415.5–2471.3**	0.63	0.06	< .001*	1.9	1.7	2.1
**2471.4–2533**	0.47	0.06	< .001*	1.6	1.4	1.8
**2533.1–2591.2**	0.4	0.07	< .001*	1.5	1.3	1.7
**2591.3–2643.7**	0.53	0.06	< .001*	1.7	1.5	1.9
**Elevation (m):**						
**-15.9–138.9(Ref.)**						
**139–371.4**	0.14	0.11	0.202	1.2	0.9	1.4
**371.5–707.1**	0.35	0.11	0.001	1.4	1.2	1.7
**707.2–1154.7**	0.53	0.1	< .001*	1.7	1.4	2.1
**1154.8–2179**	0.5	0.09	< .001*	1.7	1.4	2
**Slope:**						
**0.1–6.2(Ref.)**						
**6.3–12.8**	0.14	0.07	0.067	1.1	1	1.3
**12.9–20**	0.09	0.08	0.25	1.1	0.9	1.3
**20.1–79.6**	0.11	0.06	0.07	1.1	1	1.2
**Geology:**						
**D (Ref.)**						
**C**	-0.06	0.05	0.301	0.9	0.8	1.1
**H20**	0.62	0.07	< .001*	1.9	1.6	2.1
**JK, P, Q, S, T,Tr**	-0.04	0.04	0.311	1	0.9	1
**Curvature:**						
**(-37.5)- 2 (Ref.)**						
**(2.1–42.6)**	0.003	0.05	0.941	1	0.9	1.1
**Flow Accumulation:**						
**0.1–1169346.8(Ref.)**						
**1169346.9–27107584**	-0.02	0.04	0.578	1	0.9	1.1
**Flow Direction:**						
**1.001–2(Ref.)**						
**2.001–128**	0.09	0.05	0.099	1.1	1	1.2
**Distance (m):**						
**0.1–1(Ref.)**						
**1.1–3216**	-0.04	0.04	0.292	1	0.9	1
**LULC:**						
**Flooded vegetation (Ref.)**						
**Water/ Crops/Built Area/Bare ground /Rangeland**	0.22	0.27	0.419	1.2	0.7	2.1
**Trees/Forest**	-0.74	0.11	< .001*	0.5	0.4	0.6

Note. Estimates represent the log odds of "susceptibility = 1" vs. "susceptibility = 2”.

*Statically significant.

The study found that higher levels of annual rainfall are associated with an increased risk of flooding, showing a strong correlation between increased rainfall and heightened flood hazard. Each level of rainfall showed a positive coefficient with a statistically significant p-value (<0.001), resulting in odds ratios ranging from 1.5 to 1.9. This suggests that areas with higher annual rainfall are 1.5 to 1.9 times more likely to experience floods compared to the average category.

Elevation also plays a role (p-value < 0.001). Lower elevations (139m - 371.4m) have a slightly positive coefficient, indicating a possible association with higher flood risk. Higher elevations show a clear trend. Locations between 371.5 m and 2,179 m have progressively higher coefficients and significant p-values, indicating a decreasing risk of floods with increasing elevation. Odds ratios range from 1.4 to 1.7, suggesting these areas are 1.4 to 1.7 times less likely to flood compared to the reference category. There’s a slight trend towards higher odds with steeper slopes, but the confidence interval includes 1.0 for all categories, indicating minimal influence. Steeper slopes (6.3 m—12.8 m) have a positive coefficient, indicating an association with floods.

Geology categories C, JK, P, Q, S, T, and Tr, except water bodies (H2O) don’t significantly affect flood susceptibility compared to the reference category based on the coefficients and non-significant p-values (p-values > 0.05). Water bodies have a strong positive influence (p-value < 0.001), increasing the odds of flooding by a factor of 1.9. The study area was primarily defined by linear and convex curvature classes, but they were not found to be a significant predictor of flood susceptibility according to the statistical analysis (p-values > 0.05).

The flow accumulation category estimate (1169346.9–27107584) covered a significant area within the study region, suggesting that this occurrence is not statistically significant (p-values > 0.05). Therefore, it can be inferred that it does not have a significant influence on flood vulnerability compared to the reference range. For flow direction ranging from 2.001 to 128, the coefficient of 0.09 is positive; nevertheless, its impact is not deemed statistically significant (p-values > 0.05). This indicates that within this range, this factor does not exert a substantial influence on flood susceptibility. The negative estimate suggests that flood-prone areas are less likely as distance increases, indicating shorter distances are more prone to flooding. However, the p-value shows this relationship is not statistically significant (p = 0.292). Therefore, it can be concluded that distance does not have a significant impact on flooding likelihood for this study area.

**Fig 4:**
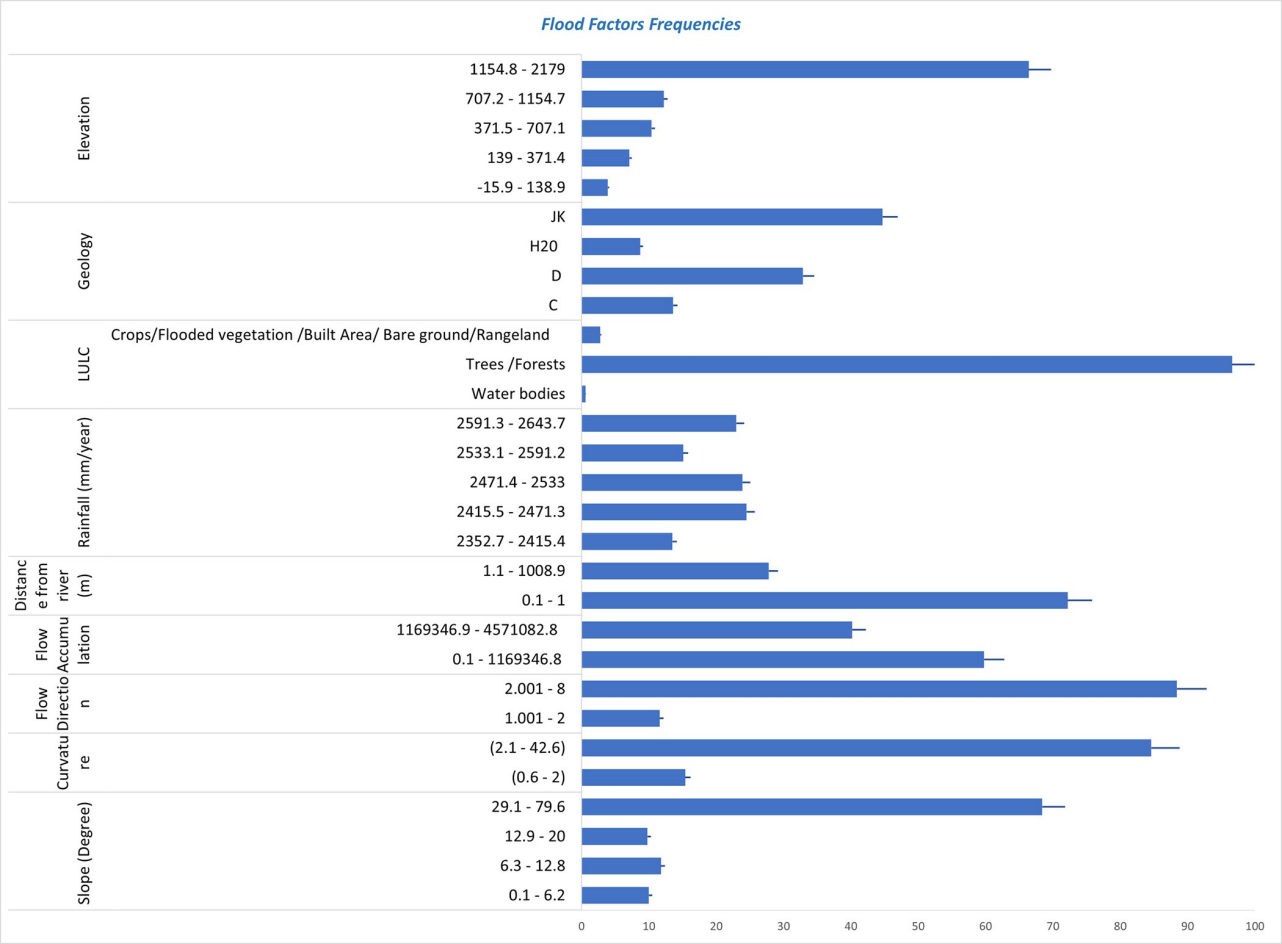
Flood factors frequency graph.

The coefficient for ’waterbodies water/ crops/built area/ bare ground /rangeland’ (0.22) indicates a positive relationship. The odds ratio (OR) is 1.2, and the confidence interval falls between 0.7 and 2.1, implying a potential rise in flood odds for water bodies in comparison to the baseline value. However, this effect does not reach statistical significance as indicated by p-values greater than 0.05. The negative coefficient (-0.74) assigned to the ’Trees/Forest’ categories signifies a negative correlation with flood susceptibility. The p-value (<0.001) demonstrate statistical significance, indicating a robust association between regions containing trees/forest and a decreased probability of experiencing floods in comparison to areas with flooded vegetation. The odds ratio (OR) of 0.5, accompanied by a confidence interval spanning from 0.4 to 0.6, further supports the notion that the presence of trees/forest significantly diminishes the likelihood of floods when contrasted with flooded vegetation “Table 1” and (Fig 4) and S2 Dataset.

### Feature selection analysis

**Fig 5:**
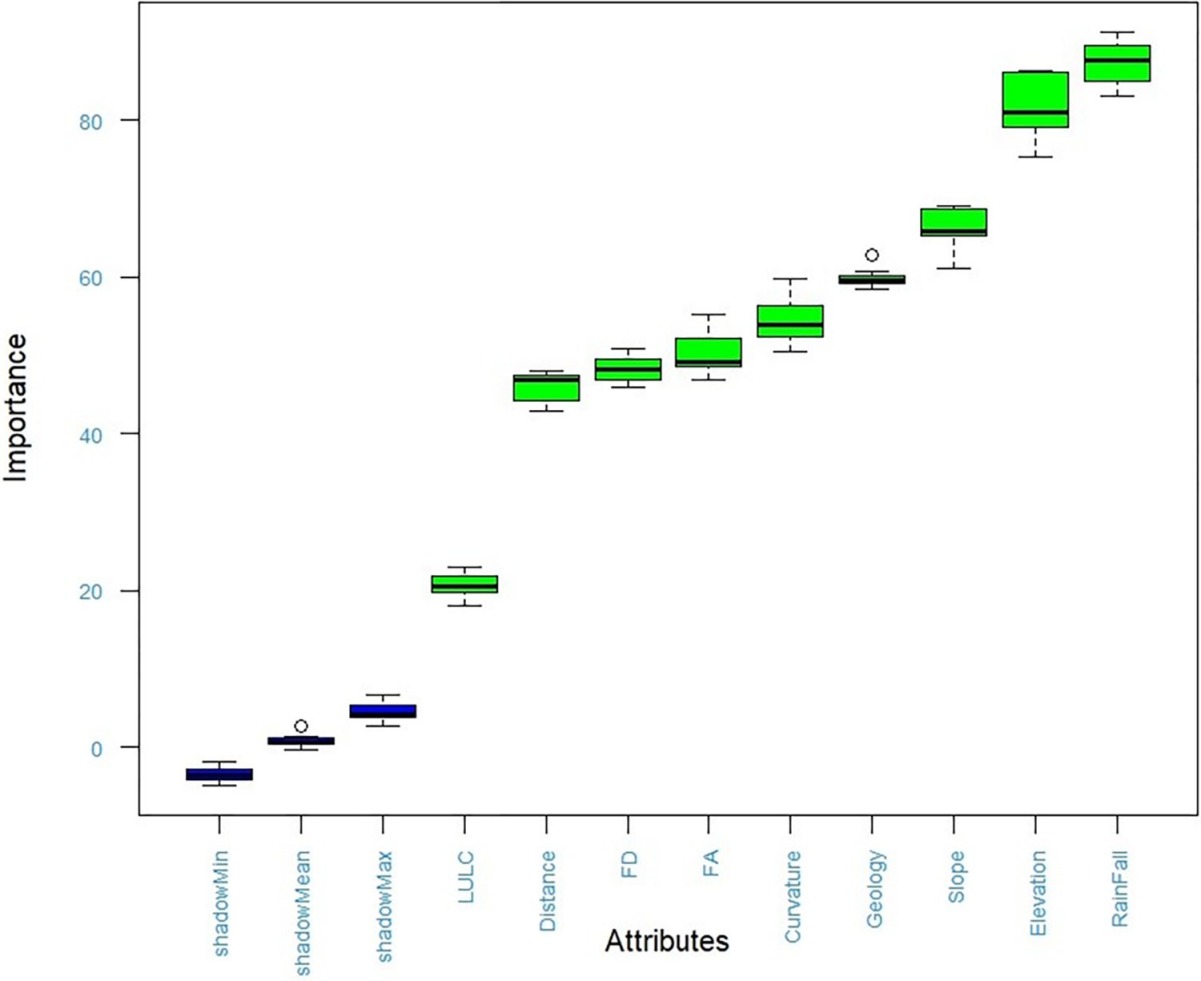
Feature importance boxplot. Blue box plots correspond to minimal, average and maximum Z score of a shadow attribute. Green box plots represent Z scores of confirmed attributes.

The Boruta algorithm, a powerful feature selection algorithm based on the Random Forest model, was utilized to analyze feature selection rankings [33]. It employs an "all-relevant" approach to identify all features significantly correlated with the target variable, rather than just a subset. Boruta introduces randomness to the dataset by generating shadow features from the original features, then uses the extended dataset to train a random forest classification model and assess the importance of each feature. The algorithm compares the importance of each original feature to the importance of its corresponding shadow feature, rejecting features that consistently show lower importance than their shadow features and marking features that have shown importance but not consistently enough to be confirmed as tentative. Boruta is a valuable tool for feature selection, particularly in dealing with complex datasets and aiming to capture all relevant information. [33,34].

(Fig 5) illustrates the relative importance of nine attributes in relation to shadow attributes. In this boxplot, the blue boxplots represent the shadow attributes, which correspond to the minimum, average, and maximum Z scores of a shadow attribute. Shadow attributes are essentially randomized versions of the original features and are utilized as a baseline for comparison. When the importance score of a genuine attribute consistently exceeds that of the shadow attributes, it is indicative of its significance. Ideally, the importance scores of shadow attributes should approach zero. The boxplot includes three blue representations for the minimum, mean, and maximum attributes. Different colors within the boxplots are employed to convey decisions regarding each feature: ’Green’ signifies that the feature is confirmed as important, ’Red’ denotes that the feature is deemed unimportant, and ’Yellow’ indicates that the feature’s importance remains uncertain [35].

Based on the findings presented in (Fig 5), it is evident that all nine attributes are considered significant, as indicated by the green color of the feature boxplots. None of the attributes are regarded as unimportant or uncertain. The median importance values for rainfall, elevation, slope, geology, curvature, flow accumulation, flow direction, distance, and LULC are reported as 94.73, 87.12, 68.76, 64.04, 56.27, 52.03, 51.33, 49.43, and 21.40, respectively, as shown in (Fig 5).

**Fig 6:**
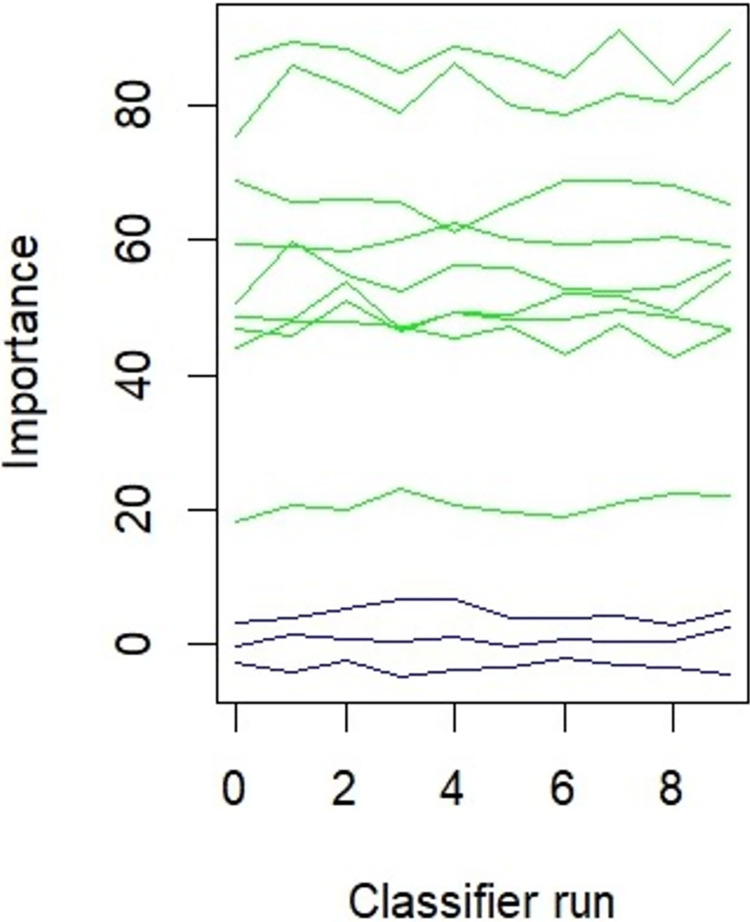
Feature selection: Classifier run.

Fig 6 illustrates the classifier run plot generated by the Boruta algorithm, depicting the progression of feature importance across various iterations. The horizontal axis denotes the number of classifier runs (iterations), while the vertical axis indicates the importance score assigned to each feature. Each line in the plot represents a distinct feature, encompassing both original and shadow features. The features are typically color-coded to reflect their final classification status: green signifies confirmed important features, yellow denotes tentative features (with uncertain importance), and red indicates features that have been rejected as unimportant. A steady upward trend in a line suggests that a feature is gaining importance over time, implying its relevance to the target variable, whereas a flat or declining line may indicate that the feature is less significant or potentially redundant. A feature whose line consistently remains above that of the shadow features is considered a strong candidate for selection, while a line that overlaps with shadow features suggests that the feature may not be significantly different [36].

In our research, as illustrated in Fig 6, all feature lines are depicted in green and exhibit a consistent upward trend, thereby affirming their significance. Furthermore, each feature line remains distinctly above the shadow features without any overlap, suggesting their strong candidacy for selection (Fig 6) and S2 File.

The analysis of feature selection ranking revealed that the main factors responsible for flooding in the study area are rainfall and elevation. Additionally, other significant attributes that contribute to flooding include slope, geology, curvature, flow accumulation, flow direction, distance from the river, and land use/land cover (LULC) patterns. The classifier runs also indicated that all nine flood influencing factors were validated characteristics, with no tentative or rejected features identified. Therefore, this study incorporated all these nine variables to model flood susceptibility (Figs 5 and 6) and S2 File.

### Flood susceptibility map

The flood susceptibility maps were created using the ArcGIS software (ESRI ArcGIS Pro v3.0.1 x64). Spatial statistics techniques were employed to produce the susceptibility map. Initially, flood inventory and thematic maps (elevation, slope, curvature, flow direction, flow accumulation, distance from river, rainfall, land-use, and geology) were developed using the ArcGIS software (ESRI ArcGIS Pro v3.0.1 x64). The raster datasets of the flood influencing factors were reclassified within the GIS environment. Subsequently, flood susceptibility maps were derived from the reclassified pixel values of the raster datasets of the nine flood influencing factors using the ArcGIS software (ESRI ArcGIS Pro v3.0.1 x64). To generate the flood susceptibility map, random forest-based boosted classification and regression algorithms were utilized. Predictions for categorical variables were made using explanatory variables from attribute tables of the raster datasets of the flood influencing factors. The models were trained using 70% of the data and validated using the remaining 30%. The input data utilized for training included the ’Pahang Point’ shape file data generated within the ArcGIS platform, along with explanatory raster data containing flood influencing variables. The x-longitudinal, and y-latitudinal areas of the ’Pahang Point’ shape file were calculated by using the special statistical tool. The analysis incorporated a sample of 100 trees, with 5 randomly selected variables under consideration. In order to validate the model’s accuracy, 30% of the training data was set aside. The flood susceptibility map values were classified into five categories: very high (Pixel values: 945277.4–984978.2), high (Pixel values: 919961–945277.3), moderate (Pixel values: 895795.2–919960.9), low (Pixel values: 869903.4–895795.1), and very low (Pixel values: 838257.8–869903.3), using the natural break classification method.

**Fig 7:**
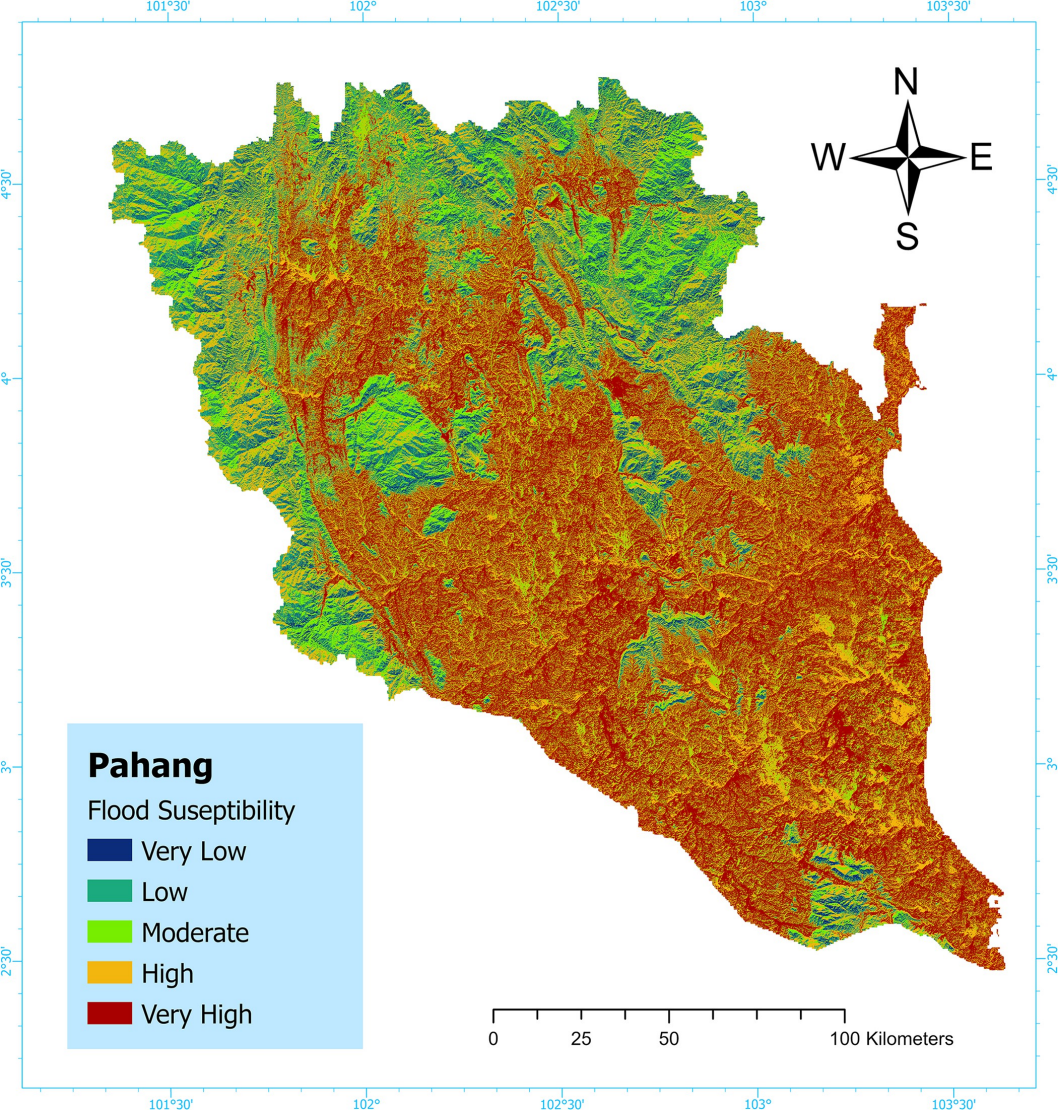
Flood susceptibility map: Pahang. (This map has been created by the author using ArcGIS Software: ESRI ArcGIS Pro v3.0.1 x64).

The Susceptibility map indicates that the study area is predominantly characterized by the very high susceptibility class, covering 37.1% of the total area. Conversely, the high, moderate, low, and very low susceptibility classes account for approximately 26.3%, 20.9%, 8.0%, and 7.7% of the area, respectively [37,38] (Fig 7) and S3 Dataset.

### Validation

**Fig 8:**
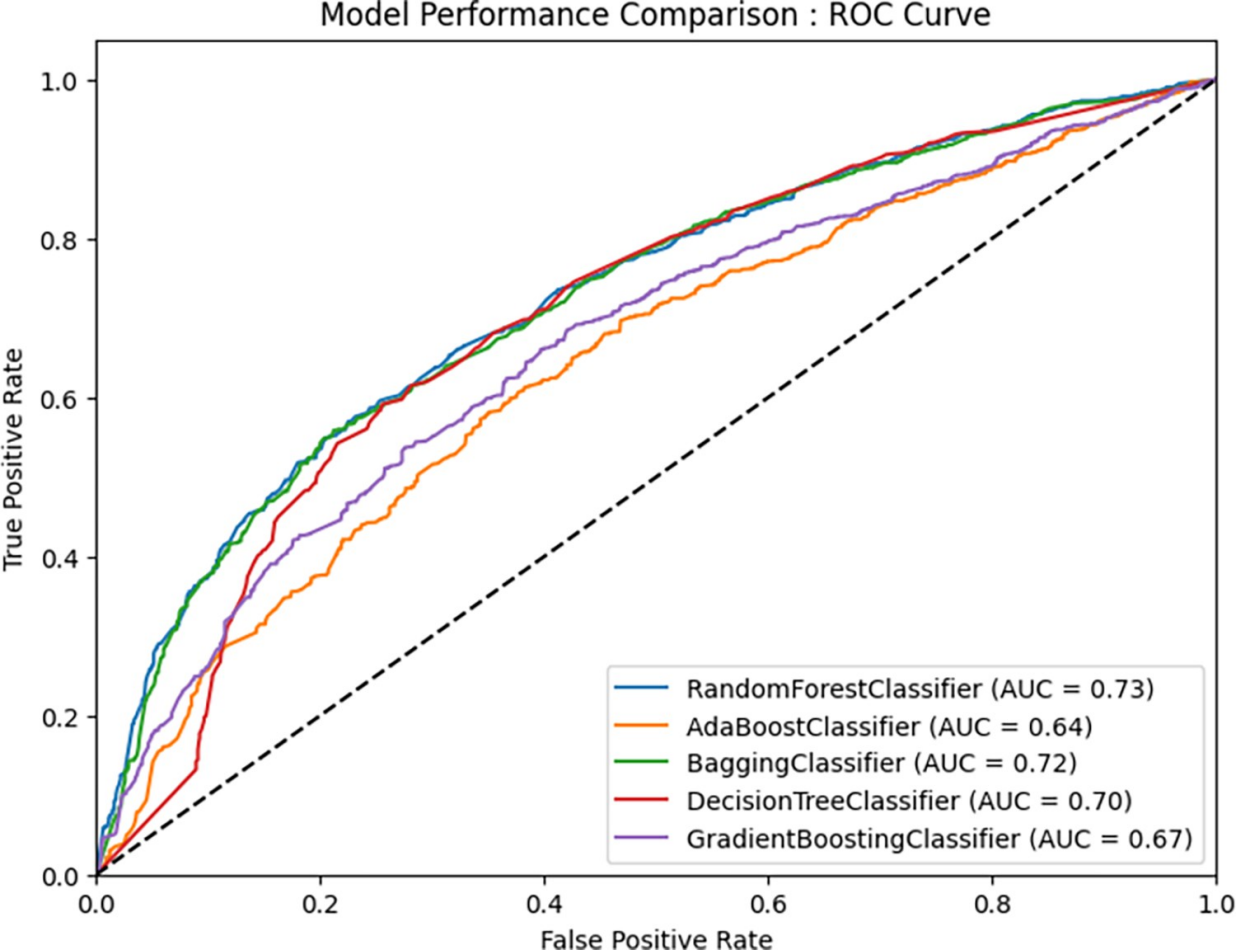
Model validation: ROC.

Fig 8 illustrates a comparative analysis of the performance of five distinct classification models: Random Forest Classifier, AdaBoost Classifier, Bagging Classifier, Decision Tree Classifier, and Gradient Boosting Classifier. The evaluation metric employed is the Receiver Operating Characteristic (ROC) Curve, which effectively depicts the balance between the true positive rate (sensitivity) and the false positive rate (1—specificity) as the classification threshold is modified. Each model’s ROC curve is supplemented by its corresponding Area Under the Curve (AUC) value. A higher AUC signifies superior performance, indicating that the model is more adept at accurately differentiating between positive and negative classes. The configuration of each ROC curve illustrates the variations in model performance as the classification threshold is altered. The x-axis denotes the False Positive Rate (FPR), representing the fraction of negative instances misclassified as positive, while the y-axis indicates the True Positive Rate (TPR), which reflects the proportion of positive instances accurately classified as positive. Ideally, one seeks a model whose ROC curve closely adheres to the top-left corner, signifying elevated TPR (sensitivity) and diminished FPR (1—specificity). A more pronounced curve near the top-left corner suggests enhanced class discrimination. Conversely, a classifier that operates purely at random would exhibit an ROC curve that aligns with the diagonal line (y = x), resulting in an AUC of 0.5. [39,40].

This research indicates that the Random Forest Classifier exhibits the highest AUC value, exceeding 0.73, which implies it may be the most effective model among those evaluated, demonstrating an accuracy greater than 71%. Following this, the Bagging Classifier, Decision Tree Classifier, and Gradient Boosting Classifier present AUC values of 0.72, 0.70, and 0.67, respectively. The AdaBoost Classifier, with the lowest AUC of 0.64, suggests a comparatively diminished performance. An AUC value greater than 0.73 is indicative of a strong score, highlighting the model’s potential reliability for real-world applications [41]—(Fig 8).

## Discussion

### The flood influencing factors

An integrated statistical EML and GIS spatial approach was used to create flood susceptibility map for the study area using the ArcGIS software (ESRI ArcGIS Pro v3.0.1 x64). Nine flood influencing factors, including elevation, slope, curvature, flow direction, flow accumulation, distance from river, rainfall, land-use, and geology, were considered. Feature selection prioritized factors influencing flooding, with rainfall and elevation identified as the most important predictors, followed by slope, geology, curvature, flow accumulation, flow direction, distance from river, and LULC. The results underscore the notable influence of these variables on the occurrence of floods within the research site.

The study findings indicated that precipitation plays a crucial role in influencing floods (p-value < 0.001). The yearly precipitation varies from 2,352.7 mm to 2,643.7 mm [Mean ± SD (2567.9 ± 59.3)], with higher precipitation being closely linked to elevated flood hazards. The results align with prior research that underscores the importance of rainfall patterns in increasing the risk of flooding in Pahang. [42,43]. The analysis of elevation data in Pahang revealed significant factors contributing to flooding (p-value < 0.001). Low-lying regions (139 m to 371.4 m) showed a positive coefficient, indicating a correlation with higher flood risk. This is due to poor drainage in low-lying areas, leading to water accumulation and flooding during heavy rainfall or overflowing water bodies. Obstructed drains can worsen flooding, even during moderate rainfall. Coastal lowlands are at higher risk due to rising sea levels from climate change, causing flooding even during normal tides. These findings support previous studies linking low-lying areas to increased flood vulnerability [44–46].

This study found that areas with steeper and convex surfaces are more prone to flooding. A surface that slopes upwards is denoted by a negative value, while a surface that curves upwards concavely is indicated by a positive profile. A surface is flat when its value is 0. The curvature of a surface influences the velocity at which fluid moves across it [47]. The presence of steep slopes can heighten the risk of flooding as they restrict the capacity of rainwater to infiltrate the ground, resulting in swift runoff. The increased velocity of water on these inclines can overpower drainage systems, resulting in flooding in lower-lying regions. Moreover, the concentrated flow from convex slopes can intensify the quantity and speed of water, further elevating the likelihood of flooding. Comparable conclusions have been observed in other research studies [5,48,49]. In this study, it was found that water bodies significantly contribute to flooding in the study area (p-value < 0.001). The presence of water bodies in Pahang leads to overflow during heavy rainfall, contributing to flooding. The study area also benefits from a stream drainage network and lowlands near major rivers. The geology map shows that the study areas consist mainly of JK, P, Q, and Tr soil types. However, it is important to note that soil textures indirectly affect flooding. The study area may have been dominated by shallow sea environments in the past, resulting in clay-rich soils that have low permeability to water, worsening the flooding situation [44,50–53].

The research findings revealed that most of the study locations exhibit low flow accumulation categories. Furthermore, it was observed that low flow accumulation categories do not serve as a strong indicator for predicting floods. This aligns with previous studies which have shown that areas with high flow accumulation typically face a higher risk of flooding [54,55]. Low flow accumulation could potentially lead to an increase in infiltration rate, resulting in the formation of more drainage channels that subsequently have a reduced impact on flooding within the research site [56]. The predominant flow direction in the study area is generally from the south to southeast, southwest, and northwest, but its effect on flooding is minimal, as indicated by previous research. Various factors, including low-lying topography, monsoonal rains, sedimentation in rivers, tidal influence, deforestation, and changes in land use, all contribute to diminishing the impact of flow direction on flooding in Pahang [57–59]. The likelihood of flooding decreases with greater distance from rivers or streams, in line with findings from previous research. This can be attributed to factors such as drier soil, reduced accumulation of debris, slower water flow, and fewer sedimentation problems [28,59].

Our research findings indicate that the presence of ’waterbodies/crops/built area/bare ground/rangeland’ has a positive impact on flooding within the study area. Conversely, our study reveals a strong correlation between areas with trees/forests and a reduced likelihood of floods compared to areas with flooded vegetation. The odds ratio (OR) is 0.5, with a confidence interval ranging from 0.4 to 0.6, suggesting that areas with trees/forests significantly decrease the probability of floods compared to areas with flooded vegetation (p-value <0.001). These findings align with previous studies that have also demonstrated the influence of LULC on flooding. One possible explanation for this phenomenon is that the extensive root systems of trees/forests create permeable soil, which absorbs and retains rainwater, preventing it from rapidly flowing into rivers and causing floods. Additionally, forests play a crucial role in preventing soil erosion by anchoring the soil with their roots, thereby reducing the amount of sediment entering rivers. This, in turn, helps maintain the rivers’ capacity and mitigates the severity of flooding. Furthermore, forests act as a barrier that slows down the flow of rainwater, further contributing to flood prevention [60–64].

### Feature selection

The research employed the feature selection technique to prioritize flood influencing factors using MDA, encompassing classification and regression methodologies [65]. FS analysis revealed that all nine variables played significant roles as flood influencing factors within the study area. Among these variables, ’rainfall’ and ’elevation’ were identified as having the highest priorities, with ’slope’, ’geology’, ’curvature’, ’flow accumulation’, ’flow direction’, ’distance from river’, and ’LULC’ following in importance. All nine flood influencing attributes are interconnected. In Pahang, Malaysia, heavy rainfall during the northeastern monsoon season from November to March is a significant factor in causing floods. The intensity of rainfall overwhelms rivers’ capacity, leading to overflow and subsequent flooding of surrounding areas. Water accumulation in low-lying regions, deforestation, and urbanization can also contribute to flooding by reducing the land’s ability to absorb rainwater and increasing surface runoff. Poor maintenance of crops and farmlands can obstruct water flow during heavy rainfall. Low-lying areas become saturated and unable to drain effectively, making them vulnerable to flooding from overflowing rivers or rising sea levels. Steeper slopes facilitate rapid downhill movement of rainwater, increasing water flow towards streams and rivers and contributing to flooding. Steeper slopes are also prone to soil erosion during heavy rainfall, depositing sediment in rivers and streams and reducing their capacity to carry water downstream. In summary, low-lying areas collect water while steeper slopes accelerate the flow of water towards these regions, ultimately leading to floods [66–69].

### The flood susceptibility map

The research conducted a mapping of flood-prone regions in Pahang by employing a GIS-based EML algorithm. To avoid overfitting to the training data, the RF classifier model was utilized. An AUC value of >0.73 on the validation datasets signified the satisfactory performance of the models. The susceptibility map shows that a significant portion of the Pahang region, specifically the Eastern, Southern, and central areas, are highly prone to flooding. This is due to various factors such as high levels of annual rainfall (2533.1–2643.7 mm/year), low-lying areas, steep and convex slopes, proximity to coastal areas and streams, and the presence of flooded vegetation, crop lands, built-up areas, bare ground, and rangeland. Conversely, the flood susceptibility map indicates that the northern, Northeast, northwest, and southern zones have a low susceptibility to flooding. These areas are characterized by abundant trees and forests, lower annual rainfall, higher elevations (707.2–2179 m) with steeper slopes, and are located further away from coastal regions. Addressing anthropogenic factors that contribute to climate change is crucial. If not addressed, climate change can lead to higher sea levels, more frequent and severe extreme weather events like floods, droughts, and wildfires, disruptions in agricultural productivity, and the extinction of many species. Human activities such as deforestation, urbanization, and converting wetlands into agricultural land increase runoff and decrease water absorption, increasing the risk of flooding. The impact of levees and drainage systems on flooding is considered when assessing flood susceptibility. Flood susceptibility maps can help communities prepare for floods by establishing early warning systems, evacuation plans, and flood-resistant infrastructure. The flood susceptibility mapping also aids in making informed decisions about land use planning to reduce flood risk. It is essential to allocate resources to prioritize flood protection efforts in the most vulnerable areas to mitigate future risks. Our results align with prior research [69–71].

Malaysia, a tropical nation, has ample water resources with an average annual precipitation of 2400 mm. However, uneven rainfall distribution leads to droughts in certain areas and floods in others. This highlights the importance of utilizing rainwater as an alternative source and implementing measures to reduce flash floods. The anticipated impact of climate change suggests a decline in future precipitation levels across multiple states in Malaysia. In Malaysia, the non-revenue water levels range from 19% in P. Pinang to 50% in Pahang across different states. Several countries, including Japan, Uganda, the USA, and Germany, offer subsidies and incentives for property owners who install Rainwater Harvesting Systems (RWHS). Malaysia could benefit from implementing a similar program to utilize its abundant rainfall and reduce flooding risks [72].

The process of flood susceptibility mapping presents numerous challenges and uncertainties. In this research, the focus was on creating a GIS-based flood susceptibility map for the study area using the EML algorithm. A unique aspect of this study was the development of an RF-embedded model for assessing flood susceptibility in the area. The primary contribution of this research is the potential improvement in establishing more accurate flood models and presenting results in a spatial context. Nevertheless, there are challenges associated with ML algorithms, such as the need for data cleaning and refinement before further processing. Additionally, extracting specific features from large datasets can be a costly endeavor. Another challenge is the possibility of attrition bias when dealing with missing values in the datasets. Despite these challenges, the RF-embedding classifier model proved to be the most effective technique for reducing bias, outperforming other models with its high accuracy, easy interpretation, and excellent prediction performance [37].

The findings of this study have the potential to greatly benefit industries that face flooding in areas prone to frequent and severe rainfall. Utilizing machine learning techniques has proven effective in addressing uncertainties surrounding flood susceptibility, especially when accurate historical flood inventory maps are accessible [65,73]. The flood susceptibility maps play a crucial role in managing risks and hazards associated with floods, as well as in mapping and assessing disasters. Our research holds immense significance across various domains. It provides national agencies with the ability to identify high-risk areas, enabling them to prioritize the allocation of resources towards flood protection infrastructure and emergency response plans. Additionally, it contributes to the development of infrastructure and the promotion of sustainable construction practices. These flood susceptibility maps also serve to raise public awareness about flood risks within their communities. Moreover, our study aids decision-makers in implementing community-level strategies to reduce risks, such as the development of evacuation plans, the establishment of flood warning systems, and the promotion of flood insurance. By identifying areas prone to flooding, policymakers can enforce land-use regulations that restrict development in high-risk zones or mandate the implementation of flood-resistant building codes. Furthermore, our research proves valuable in making informed urban planning decisions, encouraging the development of green spaces and natural drainage systems that help mitigate flood risks. Lastly, our study assists in identifying vulnerable populations within communities, allowing for targeted outreach and support during flood events.

## Conclusions

This study utilized an integrated approach combining EML algorithms with GIS to assess flood susceptibility in Pahang, Malaysia. Through the application of the Random Forest model, nine key flood influencing factors were analyzed, revealing rainfall, elevation, and slope as the most significant predictors of flood vulnerability. The generated flood susceptibility map highlighted high-risk areas in the eastern, southern, and central zones of Pahang, indicating the urgent need for targeted flood mitigation strategies in these regions. However, despite the insights gained from this research, some gaps remain to be addressed.

One notable gap lies in the limited consideration of temporal variability in flood susceptibility. Traditional flood susceptibility mapping often fails to capture the temporal changes in flood risk, which poses a significant limitation. Various solutions and methodologies can be employed to address this issue. For instance, remote sensing techniques can utilize time series data from satellites like Moderate Resolution Imaging Spectroradiometer (MODIS) to monitor changes in land cover, such as deforestation and urbanization, that affect flood risk. Additionally, historical and projected precipitation data from weather stations and climate models can be integrated to understand how rainfall patterns may impact future flood events. Furthermore, historical stream gauge data on water levels and flow rates can help identify areas with varying flood risk due to seasonal fluctuations. Machine learning algorithms can also be utilized to analyze historical data, identify patterns, and predict future flood susceptibility under changing conditions. Creating multiple flood susceptibility maps based on different climate change and land-use change scenarios can provide a range of potential future risks. Interactive mapping tools, such as web-based maps, can allow users to explore how flood risk might evolve under different scenarios. However, the effectiveness of these methods relies on the availability of historical and projected data for the specific region of interest. Moreover, complex computational resources, such as dynamic modelling and advanced machine learning approaches, may be necessary. Effective implementation of these approaches often requires collaboration among hydrologists, geographers, data scientists, and urban planners. By incorporating these solutions and methodologies, flood susceptibility maps can become more dynamic and informative, empowering communities to enhance their preparedness and management of flood risks in a changing environment. Future research endeavors should explore the incorporation of temporal dynamics, including seasonal variations, land-use pattern changes, and climate fluctuations, to enhance the predictive accuracy and applicability of flood susceptibility models over time. While this study identified important flood influencing factors, further investigation is needed to better understand the complex interactions among these variables and their implications for flood risk management.

The validation process of the flood susceptibility model revealed satisfactory performance; however, future studies could benefit from more robust validation techniques and comparisons with alternative modelling approaches to ensure the reliability and generalizability of the findings. The utilization of advanced machine learning techniques and higher-resolution datasets may offer opportunities to refine the accuracy and granularity of flood susceptibility mapping, particularly in regions with complex topography or rapidly changing environmental conditions. Although advanced machine learning techniques and higher-resolution datasets have the potential to improve flood susceptibility mapping, there are several challenges and limitations that need to be considered during their implementation. One challenge is the management of large amounts of data and the computational resources needed for processing, especially when dealing with high-resolution datasets from LiDAR or satellite imagery. The storage and computational requirements can be significant, presenting a major obstacle. Another limitation is the availability of high-quality historical data on past flood events, precipitation patterns, land-use changes, and other relevant factors. This data may not be easily accessible in all areas, which can hinder the accuracy and effectiveness of the mapping process. Additionally, training advanced machine learning techniques like deep learning is complex and requires substantial computational power and time. This can be problematic for institutions with limited resources, making it difficult for them to adopt these techniques. Furthermore, acquiring high-resolution data and the necessary computational resources for advanced machine learning can be expensive, particularly for developing countries with limited financial means. The cost associated with implementing these techniques can act as a barrier, preventing their widespread adoption in resource-constrained regions. Moreover, successfully implementing these techniques requires expertise in data science, machine learning, and flood hydrology. Unfortunately, not all planning agencies have access to such expertise, further hindering their ability to effectively utilize these advanced techniques. Additionally, it is crucial to regularly update and recalibrate the models as flood risks and environmental conditions change over time. This ongoing process requires continuous allocation of resources and expertise to ensure the accuracy and reliability of the mapping results. Partnerships between research institutions and local planning agencies can play a crucial role in addressing the challenges at hand. These partnerships can facilitate the sharing of data, the building of capacity, and the development of models. The increasing availability of open-source tools and cloud computing platforms can also contribute to this effort by alleviating the computational burden. To enhance trust in the predictions made by machine learning models, it is important to focus on interpretable models. Selecting or developing models that provide insights into their decision-making processes can increase transparency and understanding. This, in turn, can foster trust in the predictions made by these models.

While advanced techniques hold significant potential, it is essential to adopt a balanced approach that considers feasibility, practicality, and available resources. In some cases, simpler models trained on readily available data may be a more practical option, particularly in regions with limited resources. However, as technology and expertise become more accessible, advanced methods have the potential to revolutionize flood susceptibility mapping, leading to better prepared and more resilient communities.

## Supporting information

S1 File Supportive information. (XLSX)

S2 File Code Snippets. (DOCX)

S1 Dataset Dataset-Rainfall. (XLSX)

S2 Dataset (XLSX)

S3 Dataset Dataset (ascii). (DOCX)

## References

[1] Disaster | UNDRR. [cited 5 Apr 2024]. Available: https://www.undrr.org/terminology/disaster.

[2] Du W, Fitzgerald GJ, Clark M, Hou XY. Health impacts of floods. Prehospital and Disaster Medicine. Cambridge University Press; 2010. pp. 265–272. doi: 10.1017/S1049023X00008141 20586021

[3] Sun F, Lai X, Shen J, Nie L, Gao X. Initial allocation of flood drainage rights based on a PSR model and entropy-based matter-element theory in the Sunan Canal, China. Zhou G, editor. PLoS One. 2020;15: e0233570. doi: 10.1371/journal.pone.0233570 32479523 PMC7263625

[4] Khan MMA, Shaari N, Nahar A, Baten MdA, Nazaruddin DA. Flood impact assessment in Kota Bharu, Malaysia: a statistical analysis. 2014.

[5] Nurul Ashikin A, Nor Diana MI, Siwar C, Alam MdM, Yasar M. Community Preparation and Vulnerability Indices for Floods in Pahang State of Malaysia. Land (Basel). 2021;10: 198. doi: 10.3390/land10020198

[6] ASM—Academy of Sciences Malaysia. Assessment on the Sustainability of the Tasik Chini Basin and Tasik Chini Biosphere Reserve—Official Portal Academy of Sciences Malaysia. 28 Nov 2023 [cited 22 Apr 2024]. Available: https://www.akademisains.gov.my/asm-publication/assessment-on-the-sustainability-of-the-tasik-chini-basin-and-tasik-chini-biosphere-reserve/.

[7] Britannica. Malaysia—Tropical, Monsoon, Humid. In: Climate of Malaysia [Internet]. 2024 [cited 22 Apr 2024]. Available: https://www.britannica.com/place/Malaysia/Climate.

[8] DARUL MAKMUR. Portal Rasmi Kerajaan Negeri Pahang. 5 Apr 2024 [cited 5 Apr 2024]. Available: https://www.pahang.gov.my/.

[9] Saimi FM, Hamzah FM, Toriman ME, Jaafar O, Tajudin H. Trend and Linearity Analysis of Meteorological Parameters in Peninsular Malaysia. Sustainability. 2020;12: 9533. doi: 10.3390/su12229533

[10] Muhammad NS, Abdullah J, Julien PY. Characteristics of Rainfall in Peninsular Malaysia. J Phys Conf Ser. 2020;1529: 052014. doi: 10.1088/1742-6596/1529/5/052014

[11] Zhang Cha, Ma Yunqian. Ensemble Machine Learning. Ensemble Machine Learning. Springer New York; 2012. doi: 10.1007/978-1-4419-9326-7

[12] Han S, Kim H, Lee YS. Double random forest. Mach Learn. 2020;109: 1569–1586. doi: 10.1007/s10994-020-05889-1

[13] Mahajan P, Uddin S, Hajati F, Moni MA. Ensemble Learning for Disease Prediction: A Review. Healthcare (Switzerland). MDPI; 2023. doi: 10.3390/healthcare11121808 37372925 PMC10298658

[14] Parsuramka R, Goswami S, Malakar S, Chakraborty S. An Empirical Analysis of Classifiers Using Ensemble Techniques. Advances in Intelligent Systems and Computing. Springer; 2021. pp. 283–298. doi: 10.1007/978-981-15-5616-6_20

[15] Shirzadi A, Soliamani K, Habibnejhad M, Kavian A, Chapi K, Shahabi H, et al. Novel GIS based machine learning algorithms for shallow landslide susceptibility mapping. Sensors (Switzerland). 2018;18. doi: 10.3390/s18113777 30400627 PMC6263474

[16] Tahmina A. K, Siventhiran S B, Maheswaran S, Saravana Selvan, Sreeramanan S, Low J An, et al. Enhancing precision flood mapping: Pahang’s vulnerability unveiled. In: protocols.io website [Internet]. 6 Jun 2024 [cited 8 Jun 2024]. doi: 10.17504/protocols.io.kxygxyy6zl8j/v1

[17] Khoirunisa N, Ku C-Y, Liu C-Y, Esteban D, López-Gutiérrez J-S, Negro V, et al. A GIS-Based Artificial Neural Network Model for Flood Susceptibility Assessment. International Journal of Environmental Research and Public Health 2021, Vol 18, Page 1072. 2021;18: 1072. doi: 10.3390/ijerph18031072 33530348 PMC7908221

[18] Nguyen VN, Yariyan P, Amiri M, Tran AD, Pham TD, Do MP, et al. A new modeling approach for spatial prediction of flash flood with biogeography optimized CHAID tree ensemble and remote sensing data. Remote Sens (Basel). 2020;12: 1373. doi: 10.3390/RS12091373

[19] Xu K, Fang J, Fang Y, Sun Q, Wu C, Liu M. The Importance of Digital Elevation Model Selection in Flood Simulation and a Proposed Method to Reduce DEM Errors: A Case Study in Shanghai. International Journal of Disaster Risk Science. 2021;12: 890–902. doi: 10.1007/s13753-021-00377-z

[20] Earthdata. Earthdata Search Search. In: NASA. USA.gov [Internet]. 2023 [cited 6 Jul 2023]. Available: https://search.earthdata.nasa.gov/search.

[21] Chaulagain D, Ram Rimal P, Ngando SN, Nsafon BEK, Suh D, Huh JS. Flood susceptibility mapping of Kathmandu metropolitan city using GIS-based multi-criteria decision analysis. Ecol Indic. 2023;154: 110653. doi: 10.1016/j.ecolind.2023.110653

[22] Ramesh V, Iqbal SS. Urban flood susceptibility zonation mapping using evidential belief function, frequency ratio and fuzzy gamma operator models in GIS: a case study of Greater Mumbai, Maharashtra, India. Geocarto Int. 2022;37: 581–606. doi: 10.1080/10106049.2020.1730448

[23] Towfiqul Islam ARM, Talukdar S, Mahato S, Kundu S, Eibek KU, Pham QB, et al. Flood susceptibility modelling using advanced ensemble machine learning models. Geoscience Frontiers. 2021;12: 101075. doi: 10.1016/j.gsf.2020.09.006

[24] Liu X, Zhou P, Lin Y, Sun S, Zhang H, Xu W, et al. Influencing Factors and Risk Assessment of Precipitation-Induced Flooding in Zhengzhou, China, Based on Random Forest and XGBoost Algorithms. International Journal of Environmental Research and Public Health 2022, Vol 19, Page 16544. 2022;19: 16544. doi: 10.3390/ijerph192416544 36554425 PMC9779007

[25] NASA. NASA Prediction Of Worldwide Energy Resources (POWER) | Data Access Viewer Enhanced (DAVe). 2022 [cited 26 Jun 2024]. Available: https://power.larc.nasa.gov/data-access-viewer/?fbclid=IwAR1yPlfK_3RPZbL3RWwHIrizUeq8SugivFCDN7ASnIeuC8lfO-3TJSlrlRg.

[26] Khouni I, Louhichi G, Ghrabi A. Use of GIS based Inverse Distance Weighted interpolation to assess surface water quality: Case of Wadi El Bey, Tunisia. Environ Technol Innov. 2021;24: 101892. doi: 10.1016/j.eti.2021.101892

[27] Sugianto S, Deli A, Miswar E, Rusdi M, Irham M. The Effect of Land Use and Land Cover Changes on Flood Occurrence in Teunom Watershed, Aceh Jaya. Land (Basel). 2022;11: 1271. doi: 10.3390/land11081271

[28] USGS. U.S. Geological Survey. In: The Water Cycle [Internet]. 2 Oct 2022 [cited 13 Apr 2024]. Available: https://www.usgs.gov/special-topics/water-science-school/science/water-cycle.

[29] Steinshouer DW, Qiang J, McCabe PJ, Ryder RT. Maps showing geology, oil and gas fields, and geologic provinces of the Asia Pacific region. Open-File Report. 1999. doi: 10.3133/ofr97470f

[30] Brown G. Ensemble Learning. Encyclopedia of Machine Learning. Springer US; 2011. pp. 312–320. doi: 10.1007/978-0-387-30164-8_252

[31] Breiman L. Random forests. Mach Learn. 2001;45: 5–32. doi: 10.1023/A:1010933404324

[32] Netzer M, Baumgartner C, Baumgarten D. Predicting prediction: A systematic workflow to analyze factors affecting the classification performance in genomic biomarker discovery. PLoS One. 2022;17. doi: 10.1371/journal.pone.0276607 36350811 PMC9645616

[33] Masrur Ahmed AA, Deo RC, Feng Q, Ghahramani A, Raj N, Yin Z, et al. Deep learning hybrid model with Boruta-Random forest optimiser algorithm for streamflow forecasting with climate mode indices, rainfall, and periodicity. J Hydrol (Amst). 2021;599: 126350. doi: 10.1016/j.jhydrol.2021.126350

[34] Chen L, Mei Z, Guo W, Ding S, Huang T, Cai Y-D. Recognition of Immune Cell Markers of COVID-19 Severity with Machine Learning Methods. Babu S, editor. Biomed Res Int. 2022;2022: 1–12. doi: 10.1155/2022/6089242 35528178 PMC9073549

[35] Mishra D, Das BS, Sinha T, Hoque JM, Reynolds C, Rafiqul Islam M, et al. Living with arsenic in the environment: An examination of current awareness of farmers in the Bengal basin using hybrid feature selection and machine learning. Environ Int. 2021;153: 106529. doi: 10.1016/j.envint.2021.106529 33784587

[36] Janowski L, Wroblewski R, Dworniczak J, Kolakowski M, Rogowska K, Wojcik M, et al. Offshore benthic habitat mapping based on object-based image analysis and geomorphometric approach. A case study from the Slupsk Bank, Southern Baltic Sea. Science of the Total Environment. 2021;801: 149712. doi: 10.1016/j.scitotenv.2021.149712 34419903

[37] Breiman L. Random forests. Mach Learn. 2001;45: 5–32. doi: 10.1023/A:1010933404324

[38] Strobl C, Boulesteix AL, Kneib T, Augustin T, Zeileis A. Conditional variable importance for random forests. BMC Bioinformatics. 2008;9: 307. doi: 10.1186/1471-2105-9-307 18620558 PMC2491635

[39] Heagerty PJ, Lumley T, Pepe MS. Time-dependent ROC curves for censored survival data and a diagnostic marker. Biometrics. 2000;56: 337–344. doi: 10.1111/j.0006-341x.2000.00337.x 10877287

[40] Kamarudin AN, Cox T, Kolamunnage-Dona R. Time-dependent ROC curve analysis in medical research: Current methods and applications. BMC Med Res Methodol. 2017;17: 53. doi: 10.1186/s12874-017-0332-6 28388943 PMC5384160

[41] de Hond AAH, Steyerberg EW, van Calster B. Interpreting area under the receiver operating characteristic curve. The Lancet Digital Health. Elsevier Ltd; 2022. pp. e853–e855. doi: 10.1016/S2589-7500(22)00188-1 36270955

[42] Kamarudin MKA, Toriman ME, Abd Wahab N, Abu Samah MA, Abdul Maulud KN, Mohamad Hamzah F, et al. Hydrological and climate impacts on river characteristics of pahang river basin, Malaysia. Heliyon. 2023;9. doi: 10.1016/j.heliyon.2023.e21573 38058642 PMC10695850

[43] Sulaiman NH, Kamarudin MKA, Toriman ME, Juahir H, Ata FM, Azid A, et al. Relationship of Rainfall Distribution and Water Level on Major Flood 2014 in Pahang River Basin, Malaysia. EnvironmentAsia. 2017;10: 1–8. doi: 10.14456/ea.2017.1

[44] Elfithri R, Halimshah S, Abdullah MP, Mokhtar M, Toriman ME, Embi AF, et al. Pahang Flood Disaster: The Potential Flood Drivers. Malaysian Journal of Geosciences. 2017;1: 34–37. doi: 10.26480/mjg.01.2017.34.37

[45] Miguez M, Veról A, de Sousa M, Rezende O. Urban Floods in Lowlands—Levee Systems, Unplanned Urban Growth and River Restoration Alternative: A Case Study in Brazil. Sustainability. 2015;7: 11068–11097. doi: 10.3390/su70811068

[46] Su W, Ye G, Yao S, Yang G. Urban land pattern impacts on floods in a new district of China. Sustainability (Switzerland). 2014;6: 6488–6508. doi: 10.3390/su6106488

[47] Loganathan RM, Hunt GR. Analytical solutions for flow induced by a vertically distributed turbulent plume. Environmental Fluid Mechanics. 2019;19: 801–818. doi: 10.1007/s10652-019-09659-z

[48] Ab. Ghani A, Chang CK, Leow CS, Zakaria NA. Sungai Pahang digital flood mapping: 2007 flood. International Journal of River Basin Management. 2012;10: 139–148. doi: 10.1080/15715124.2012.680022

[49] Nor Diana MI, Chamburi S, Mohd Raihan T, Nurul Ashikin A. Assessing local vulnerability to climate change by using Livelihood Vulnerability Index: Case study in Pahang region, Malaysia. IOP Conference Series: Materials Science and Engineering. Institute of Physics Publishing; 2019. doi: 10.1088/1757-899X/506/1/012059

[50] Gao S. Geomorphology and sedimentology of tidal flats. Coastal Wetlands: An Integrated Ecosystem Approach. Elsevier; 2018. pp. 359–381. doi: 10.1016/B978-0-444-63893-9.00010–1

[51] Pacheco FAL. Regional groundwater flow in hard rocks. Science of the Total Environment. 2015;506–507: 182–195. doi: 10.1016/j.scitotenv.2014.11.008 25460951

[52] Tan Boon Kong, Komoo I. Urban geology: Case study of Kuala Lumpur, Malaysia. Eng Geol. 1990;28: 71–94. doi: 10.1016/0013-7952(90)90034-X

[53] Wahab NA, Kamarudin MKA, Toriman ME, Juahir H, Samah MAA, Azinuddin M, et al. The Assessment of Sedimentation Problems in Kenyir Hydropower Reservoir, Malaysia. Water (Basel). 2023;15: 2375. doi: 10.3390/w15132375

[54] Dawod GM, Mirza MN, Al-Ghamdi KA. GIS-Based Spatial Mapping of Flash Flood Hazard in Makkah City, Saudi Arabia. Journal of Geographic Information System. 2011;03: 225–231. doi: 10.4236/jgis.2011.33019

[55] Elkhrachy I. Flash Flood Hazard Mapping Using Satellite Images and GIS Tools: A case study of Najran City, Kingdom of Saudi Arabia (KSA). Egyptian Journal of Remote Sensing and Space Science. 2015;18: 261–278. doi: 10.1016/j.ejrs.2015.06.007

[56] Gasim MB, Toriman ME, Idris M, Lun PI, Kamarudin MKA, Nor Azlina AA, et al. River flow conditions and dynamic state analysis of Pahang river. Am J Appl Sci. 2013;10: 42–57. doi: 10.3844/ajassp.2013.42.57

[57] METMalaysia—Utama. Jabatan Meteorologi Malaysia. In: LAMAN WEB RASMI [Internet]. 13 Apr 2024 [cited 13 Apr 2024]. Available: https://www.met.gov.my/index.html.

[58] Rosmadi HS, Ahmed MF, Bin Mokhtar M, Lim CK. Reviewing Challenges of Flood Risk Management in Malaysia. Water (Basel). 2023;15: 2390. doi: 10.3390/w15132390

[59] Yi Y, Liu S, Zhang X, Yang Y, Zhu Y, Cui F, et al. Spring floods and their major influential factors in the upper reaches of Jinsha River basin during 2001–2020. J Hydrol Reg Stud. 2023;45: 101318. doi: 10.1016/j.ejrh.2023.101318

[60] Akomolafe GF, Rosazlina R. Land use and land cover changes influence the land surface temperature and vegetation in Penang Island, Peninsular Malaysia. Sci Rep. 2022;12. doi: 10.1038/s41598-022-25560-0 36481770 PMC9732054

[61] Bhattacharjee K, Behera B. Does forest cover help prevent flood damage? Empirical evidence from India. Global Environmental Change. 2018;53: 78–89. doi: 10.1016/j.gloenvcha.2018.09.004

[62] Handayani W, Chigbu UE, Rudiarto I, Surya Putri IH. Urbanization and increasing flood risk in the Northern Coast of Central Java-Indonesia: An assessment towards better land use policy and flood management. Land (Basel). 2020;9: 343. doi: 10.3390/LAND9100343

[63] Khor JF, Lim S, Ling VL, Ling L. Assessing the Impact of Deforestation on Decadal Runoff Estimates in Non-Homogeneous Catchments of Peninsula Malaysia. Water (Basel). 2023;15: 1162. doi: 10.3390/w15061162

[64] Rahayu R, Mathias SA, Reaney S, Vesuviano G, Suwarman R, Ramdhan AM. Impact of land cover, rainfall and topography on flood risk in West Java. Natural Hazards. 2023;116: 1735–1758. doi: 10.1007/s11069-022-05737-6

[65] Bolón-Canedo V, Alonso-Betanzos A. Ensembles for feature selection: A review and future trends. Information Fusion. 2019;52: 1–12. doi: 10.1016/j.inffus.2018.11.008

[66] De Bruijn KM. Resilience indicators for flood risk management systems of lowland rivers. International Journal of River Basin Management. 2004;2: 199–210. doi: 10.1080/15715124.2004.9635232

[67] Chan N. Increasing Flood risk in Malaysia: causes and solutions. Disaster Prev Manag Int J. 1997;6: 1–16.

[68] J Suhaila WMDWZAJ. Trends in Peninsular Malaysia rainfall data during Southwest monsoon and Northeast monsoon season. Sains Malays. 2010;39: 533–542.

[69] Safiah Yusmah MY, Bracken LJ, Sahdan Z, Norhaslina H, Melasutra MD, Ghaffarianhoseini A, et al. Understanding urban flood vulnerability and resilience: a case study of Kuantan, Pahang, Malaysia. Natural Hazards. 2020;101: 551–571. doi: 10.1007/s11069-020-03885-1

[70] Islam MM, Amir AA, Begum RA. Community awareness towards coastal hazard and adaptation strategies in Pahang coast of Malaysia. Natural Hazards. 2021;107: 1593–1620. doi: 10.1007/s11069-021-04648-2

[71] Ruiz Estrada MA, Koutronas E, Tahir M, Mansor N. Hydrological hazard assessment: THE 2014–15 Malaysia floods. International Journal of Disaster Risk Reduction. 2017;24: 264–270. doi: 10.1016/j.ijdrr.2017.06.005

[72] Lani NHM, Yusop Z, Syafiuddin A. A review of rainwater harvesting in Malaysia: Prospects and challenges. Water (Switzerland). MDPI AG; 2018. p. 506. doi: 10.3390/w10040506

[73] Mahdizadeh Gharakhanlou N, Perez L. Spatial Prediction of Current and Future Flood Susceptibility: Examining the Implications of Changing Climates on Flood Susceptibility Using Machine Learning Models. Entropy. 2022;24. doi: 10.3390/e24111630 36359720 PMC9689156

