# Supplementary material for: Enhancing precision flood mapping: Pahang’s vulnerability unveiled
Source: PLoS One. 2024 Nov 7;19(11):e0310435. doi: 10.1371/journal.pone.0310435 (PMC11542787; doi:10.1371/journal.pone.0310435)
Supplement: S2 File — (DOCX) [file pone.0310435.s002.docx]

**Code snippets**

install.packages("dplyr", dependencies = TRUE)

install.packages("rabd", dependencies = TRUE)

library(dplyr)

library(randomForest)

library(caret)

library(Boruta)

library(mlbench)

data.frame(CombPG)

str(CombPG)

summary(CombPG)

set.seed(111)

boruta <- Boruta(Trained ~ ., data = CombPG, doTrace = 2, maxRuns= 77)

print(boruta)

plot(boruta, las= 2, cex.axis = 0.65, col.axis = 4)

plotImpHistory(boruta)
