# Supplementary material for: Enhancing precision flood mapping: Pahang’s vulnerability unveiled
Source: PLoS One. 2024 Nov 7;19(11):e0310435. doi: 10.1371/journal.pone.0310435 (PMC11542787; doi:10.1371/journal.pone.0310435)
Supplement: S3 Dataset — (DOCX) [file pone.0310435.s005.docx]

Please follow the hyperlink below to access the datasets. These datasets are very large, hence, unable to upload to the system.

[S5 Dataset](https://shorturl.at/O3wPG)
